# Energy-Efficient Region Shift Scheme to Support Mobile Sink Group in Wireless Sensor Networks

**DOI:** 10.3390/s18010090

**Published:** 2017-12-30

**Authors:** Yongbin Yim, Kyong Hoon Kim, Monther Aldwairi, Ki-Il Kim

**Affiliations:** 1 Department of Informatics, Gyeongsang National University, Jinju 52828, Korea; nowon28@gmail.com (Y.Y.); khkim@gnu.ac.kr (K.H.K.); 2 College of Technological Innovation, Zayed University, P.O. Box 144534, Abu Dhabi, UAE; Monther.Aldwairi@zu.ac.ae; 3 Network Engineering and Security Department, Jordan University of Science and Technology, PO Box 3030, Irbid 22110, Jordan; 4 Department of Computer Science and Engineering, Chungnam National University, Daejeon 34134, Korea

**Keywords:** mobile sink group, geocasting, mobility support, wireless sensor networks

## Abstract

Mobile sink groups play crucial roles to perform their own missions in many wireless sensor network (WSN) applications. In order to support mobility of such sink groups, it is important to design a mechanism for effective discovery of the group in motion. However, earlier studies obtain group region information by periodic query. For that reason, the mechanism leads to significant signaling overhead due to frequent flooding for the query regardless of the group movement. Furthermore, the mechanism worsens the problem by the flooding in the whole expected area. To deal with this problem, we propose a novel mobile sink group support scheme with low communication cost, called Region-Shift-based Mobile Geocasting Protocol (RSMGP). In this study, we utilize the group mobility feature for which members of a group have joint motion patterns. Thus, we could trace group movement by shifting the region as much as partial members move out of the previous region. Furthermore, the region acquisition is only performed at the moment by just deviated members without collaboration of all members. Experimental results validate the improved signaling overhead of our study compared to the previous studies.

## 1. Introduction

Wireless sensor networks (WSNs) have been regarded as a prominent network technology for realizing intelligent applications with interaction between cyber and physical worlds. A set of small sensor devices is deployed to application fields and detects surrounding events. Sinks collect and process the event data as required by the applications [1]. In a considerable number of sensor networks applications, mobile sinks are utilized to distribute load due to the restricted energy resource or to perform their own missions such as fire extinguishment, battlefield surveillance, or habitat monitoring [2]. In addition, a number of sinks could compose a group and have geographically collective movement [3], e.g., a squad of fire fighters and a platoon of soldiers. They typically try to acquire the same data to execute communal missions.

The mobile sink groups have different aspects compared to individual mobile sinks in terms of communications. Group movement has created novel routing opportunities. Utilizing the geographical adjacency, it could effectively deliver data to them without identification of each destination one by one. Since a group usually has a certain radius of action, group region could be considered as a circle with a center point and a radius. Data can be delivered by geocast routing methods into the region [4]. It significantly reduces control overhead to identify every location where destinations are located.

In order to support mobility for such groups, it is necessary to periodically obtain positions of the group. Previous studies for mobile sink groups [5,6] exploit flooding to figure out a region including all member sinks. However, the schemes have a significant energy consumption problem due to the periodic and extensive flooding in a group region. A representative sink who discovers a region of a group queries each location of member sinks in the whole expected area where the group could be located. Each member sink that received the request replies with its own location to the representative sink. Because of that, it does not only consume a large amount of energy, but it also causes frequent retransmission due to congestion and collision around the representative sink.

Meanwhile, our observation drives new possibilities. The sink groups have an inherent feature in which members of a mobile sink group typically have similar motion patterns. They typically move toward the same point while freely swinging in a certain radius of a group region. We categorize the character of the group movement into two sub-movements: macro movement and micro movement. If the group region is discovered one time, the region could be effectively discovered hereafter by shifting the region according to the macro movement. Since every member has similar movement, we could estimate macro movement of a group by investigation of only a few members’ motion information. Some errors due to the involvement of the micro movement could also be corrected by the other members’ information.

In this paper, we propose a new scheme that discovers a group region without periodic flooding, called Region-Shift-based Mobile Geocasting Protocol (RSMGP). Focusing on the inherent feature stated previously, the proposed scheme could discover a location of a group by utilizing movement information of partial member sinks without large energy consumption. We consider that movement of a group could be depicted as movement of center point. If a sink surpasses the region that the sink belongs to, the group region is updated. Of course, estimating exact group region is not easy unless region information is given at all times. Instead of that, our scheme integrates movement patterns of members out of the region so that the calculation could approximately imitate the entire movement pattern of the group. Unlike the previous schemes that periodically figure out a location of a group, the proposed scheme is more efficient since updating location of the group is triggered only when the group moves over a certain distance.

The remainder of the paper is organized as follows: in Section 2, we briefly overview the state of the art in terms of sink mobility support. In addition, we describe limitations of previous studies and appeal necessity of the proposed scheme, RSMGP. Then, Section 3 presents the network model and basic idea, and considerations in detail for solidity of our RSMGP. The proposed scheme is evaluated through experimental results in Section 4. Finally, Section 5 concludes the paper.

## 2. Related Work

**Figure 1:**
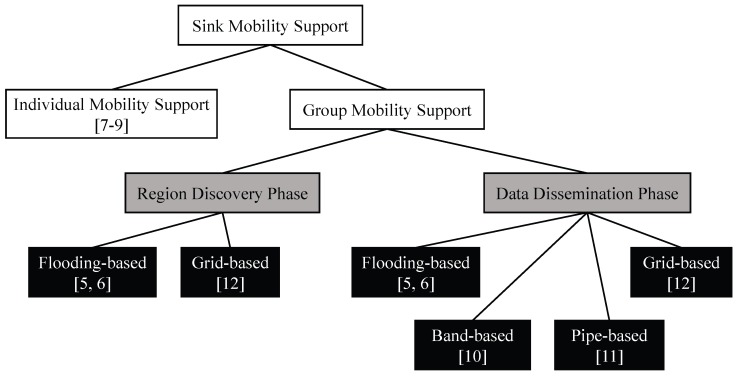
Taxonomy of sink mobility support schemes.

In order to support multiple mobile sinks, a series of schemes have been suggested. These studies can be divided into two primary types: individual and group mobility support as shown in Figure 1. In addition, group mobility support schemes could be divided into two detailed phases. Then, the studies can be categorized by approaches used in each phase.

Individual mobility support schemes [7,8,9] have focused on efficient sink location management approaches. Purposes of mobile sink are various e.g., traffic balancing, data gathering, or low latency communications, and thus the research area has been extensively conducted.

SinkTrail [7] is a broadcast-based mobility support scheme. A sink broadcasts its own status information when it moves longer than a predefined distance threshold. Sensor nodes received the information update relative position (the number of hops) to the sink and repeat broadcasting to other nodes. To reduce the broadcasting overhead, if a relative position of updated information is the same as the previous one, the update is discarded at the node because farther nodes are less affected by a single movement. However, it still seems not to be unmanageable in plenty of sinks due to the status maintenance of respective ones.

Misra et al. [8] have proposed a prediction-based tracking scheme. The study analyzes the data set of sink positions and predicts a mobility pattern. With enough data sets, it could precisely predict movement plan and significantly reduce control overhead. On the other hand, it would not apply until enough of the data set is collected. In addition, it requires quite a lot of data sets to deal with mobility patterns of multiple sinks even if they have group motion patterns.

RingRouting [9] utilizes a rendezvous area of the ring shape constructed in the center of a network. Location information of mobile sinks is stored on the rendezvous structure. When a source generates data and wants to report to the sinks, sink location query and reply message are transmitted to/from the storage. The scheme also could not be effectively applied to the mobile sink group support. Conclusively, they commonly waste signaling since it does not treat redundant movement information massively generated by member sinks having the similar motion pattern with each other. It causes excessive energy consumption and traffic congestion.

Meanwhile, group mobility support schemes exploit the movement pattern for efficient communication. The studies are based on the model that a mobile sink group moves to a target point with a certain size of a group area, while member sinks freely move in the group area. Thus, sources require information of the mobile sink group (e.g., group location and size) in order to send data to the group.

In terms of that, the mobile sink group support schemes consist of two phases: group region discovery and data dissemination. Primitive schemes [5,6] have supported the sink groups by the flooding-based approach in both phases. This approach conducts group region discovery by periodic flooding. Even a representative sink query in the expected region is four times larger than the original group region size since the representative sink is not conscious of the whereabouts located itself in the actual group region. Furthermore, every member sink replies with their own location information to the representative sink. Data dissemination should also be performed by flooding in the discovered group region whenever data is generated. The solutions are intuitive and simple, but there is room for improvement.

Park et al. [10] proposed band-based geocasting to reduce the data dissemination overhead. This approach stores data on expected movement paths in advance in the form of band. Therefore, it considerably reduces data dissemination overhead since it does not need to broadcast data into the whole group region. Usually, it assumes that the movement plan of a mobile sink group is not notified in advance and thus the data band is wrapped around a mobile sink group by using virtual infrastructure such as a grid. Then, the sink group receives the data after a while passing by the band. If the movement plan is known or could be easily predicted, it could reduce the size of the band. The approach effectively works in case the sink group has continuous movement but, in case of intermittent movement of the group, special treatments should be considered.

Pipe-based geocasting [11] reduces the data dissemination overhead by storing data in the form of a pipe line along with a diameter in a group region. After formation of the pipe line, member sinks query the pipe and retrieve data from the pipe. Since the approach constructs only pipe line virtual infrastructure on the diameter, it requires less communication cost compared to the band-based approach. In addition, although the sink group has intermittent movement pattern, it does not require extra operation for data dissemination to the sink group in temporary stop. Meanwhile, it causes unnecessary communication cost since member sinks require periodic query for data retrieval.

ERMG (Energy-efficient and Reliable Mobile Group communication protocol) [12] is a grid-based mobility support scheme. ERMG handles both region discovery and data dissemination phases. Instead of flooding for control and data packets, messages are delivered via cell heads on a predefined grid structure. However, it still suffers communication overhead on large expected areas for the region discovery.

The studies mentioned above have focused on the data dissemination phase under the assumption that region discovery works well. However, studies for the region discovery are at the primitive stage, and it still suffers from periodic and large area communications. We thus suggest an efficient region discovery scheme eliminating the large communication overhead.

## 3. Efficient Mobility Support for Sink Group Utilizing Region Shift

This section presents our approach for Region-Shift-based Mobile Geocasting Protocol (RSMGP). We define and assume related side technical issues as a network model. Then, the concepts and detailed mechanisms of RSMGP are proposed.

### 3.1. Preliminaries

Prior to the design of RSMGP, it is based on the following assumptions in terms of network environment:

- We assume that the member sinks exist on an infrastructureless sensor field such as disaster areas or battlefields. Thus, member sinks can communicate only via a wireless sensor network.
- The proposed scheme relies on position-based routing. Location update of groups and data delivery to the region are performed by this routing method.
- Each member sink is aware of its own location by global positioning system (GPS) or localization techniques [13].
- Source nodes obtain a location of a sink group by location service schemes [14].

We define a group region as a circle with a center point and a fixed radius. We refer to this region as a defined group region (DGR). We assume that member sinks in a group have the defined group region information at the initial stage by pre-programmed data or manual operation of a network administrator. Henceforth, movement of the group can be expressed as alterations of a center point. However, sensor nodes and member sinks could not be aware of the exact center point of moved DGR. The mobility support of mobile sink group thus involves the region discovery phase to figure out the (approximate) center point. Intuitively, frequent querying for locations of member sinks is a simple solution, but this behavior causes large communication overheads as mentioned above.

**Figure 2:**
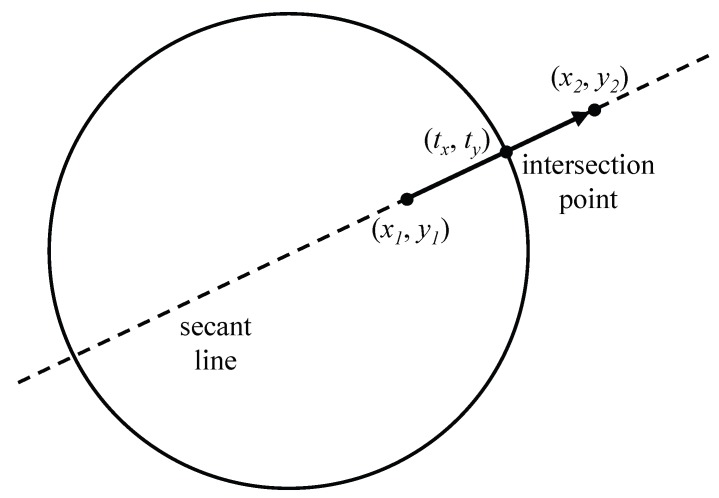
Circle and secant line.

To eliminate the frequent querying, the region shift mechanism of RSMGP utilizes deviation distance from a group region. The distance could be obtained by the property of circle–line intersection. If a member sink moves out of a group region, a (infinite) line is determined by points of before and after the movement, which is known as a secant line [15]. The secant line may intersect a determined circle as the group region as shown in Figure 2. We use the line segment from the intersection point to the point of after motion as a moving distance of the group. Thus, we need to be aware of the intersection point location. First, the secant line can be formulated as follows:

(1) $$y=\frac{y_{2}-y_{1}}{x_{2}-x_{1}}x+K,$$

where *K* is obtained by substitution of *x* and *y* as $\left(x_{1},y_{1}\right)$ or $\left(x_{2},y_{2}\right)$. In addition, the circle can be expressed as

(2) $$\begin{array}{c} {\left(x-C_{x}\right)}^{2}+{\left(y-C_{y}\right)}^{2}=R^{2},\;\\ y=\pm \sqrt{R^{2}-{\left(x-C_{x}\right)}^{2}}+C_{y}, \end{array}$$

where $\left(C_{x},C_{y}\right)$ is a center point and *R* is a radius of the circle. Then, we can obtain the intersection point of the line and circle by the simultaneous equations as follows:

(3) $$\frac{y_{2}-y_{1}}{x_{2}-x_{1}}t_{x}+K=\pm \sqrt{R^{2}-{\left(t_{x}-C_{x}\right)}^{2}}+C_{y}.$$

Consequently, two $t_{x}$ values might be derived and the one is determined by the range, $\left[x_{1},x_{2}\right]$. The line between ($t_{x}$, $t_{y}$) and ($x_{2}$, $y_{2}$) is used as the deviation distance to shift the center point.

### 3.2. Group Region Discovery by Region Shift Mechanism

As mentioned above, it is practically impossible to trace exact ideal group movements (DGR) by sensing behavior of member sinks. Previous studies provide snapshots of the group region for a certain period to approximate the movement. It collects positions of all member sinks by the flooding and calculate an average position of them. Then, the region is presented as a circle with the position and a radius covering all members. In order to overcome the shortages in terms of the communication overhead, RSMGP conducts the region discovery by our region shift mechanism. Mobile sink groups have the collective movement property. It represents motion information of one member sink implying entire movement of the group. Using this property, the proposed scheme calculates a new region location based on updates of only a few member sinks that deviate from the current region rather than all member sinks performing updates.

Though estimation of accurate movement could not be achieved, RSMGP could provide secure snapshots by utilizing the movement information of partial members and gradual correction. If an initial group region is given, some member sinks may surpass boundary of the region after a lapse of a certain amount of time. Then, the region shift process is triggered and it moves the center point location as much distance as the sinks move. In addition, region size is in accordance with a radius of DGR. Thus, we could eliminate region-wide flooding to obtain group region information by this local triggering.

**Figure 3:**
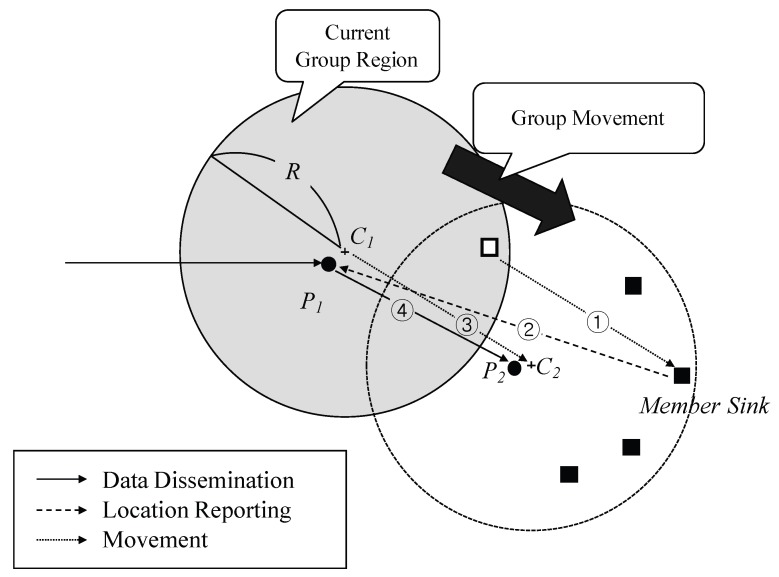
The region shift procedure.

In Figure 3, for example, the group moves from the location of a shaded circle to that of an empty circle. A member sink deviates to the previous group region. The sink reports its own location to pivot $P_{1}$ in order to notify that the group is moving. The tracing process is triggered in this way. In addition, because one or more sinks can move out of the region, it needs to calculate a new location of the sink group by combining movement information of all of them, to be discussed specifically in Section 3.4. Someone has to control these kinds of operations for region information updates. Thus, we designate the nearest node to a center point as a *pivot* node.

**Figure 4:**
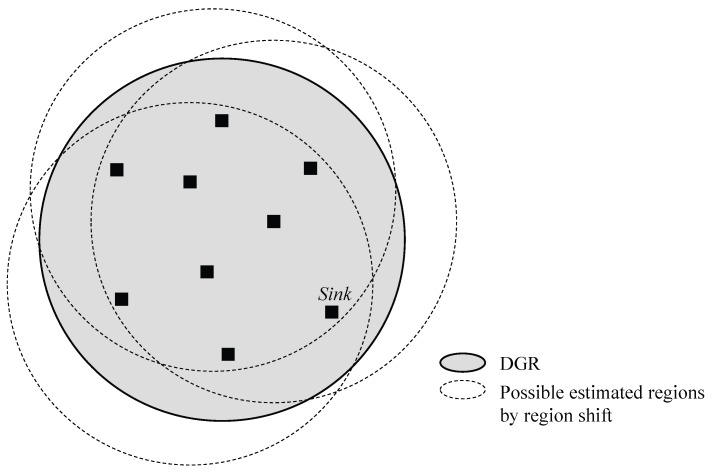
Relation between DGR and estimated region.

Member sinks can recognize whether it is within the region itself because they have region information, a center point *C* and radius *R*. Once a sink which is out of the region reports moving information to the *pivot*, this *pivot* calculates a new group location using this information. The basic algorithm of the region shift is illustrated in Algorithm 1. As shown in Figure 3, following the arrow ①, the movement of the sink group makes a sink to be out of the region firstly. ② The sink recognizes that movement out of the region and performs a location update to current pivot $P_{1}$. ③ $P_{1}$ calculates a new location $C_{2}$ of the group. $P_{1}$ announces the region information in the new region. ④ At this time, since a source is aware that the group exists in the previous location, it delivers the next data to the location $C_{1}$. When nodes in the previous region receive the data, it resets destination location to the new location and forwards it there. Figure 4 shows the relation between ideal DGR and estimated region by the region shift algorithm. Although RSMGP could not find an exact location of DGR, it provides well-deserved approximation covering all member sinks by the simple method.
**Algorithm 1** Basic algorithm of region shift at a pivot node.
1:$MMI_{p}$: member sink movement information set of pivot node *p*  2:$c_{prev}$: previous center point coordinates of a group region  3:$c_{new}$: new center point coordinates of a group region  4:*r*: radius of a group region  5:Dist(a,b): euclidean distance between geographic position *a* and *b*6:$MMI_{p}\leftarrow$ collect movement information during a certain time since the first movement report 7:**for all** $i\in MMI_{p}$ **do**8:    **if** ${\left(i\cdot x_{2}-c_{prev}\cdot x\right)}^{2}+{\left(i\cdot y_{2}-c_{prev}\cdot y\right)}^{2}>r^{2}$ **then**  ▹ if *i*’s movement deviates the previous region  9:        $c_{new}\cdot x\leftarrow c_{prev}\cdot x+Dist\left(i\cdot t_{x},i\cdot x_{2}\right)$  10:        $c_{new}\cdot y\leftarrow c_{prev}\cdot y+Dist\left(i\cdot t_{y},i\cdot y_{2}\right)$  11:        $c_{prev}\leftarrow c_{new}$  12:    **end if** 13:**end for** 14:broadcast the new region information tuple <$c_{new},r$> into the region such that ${\left(x-c_{new}\cdot x\right)}^{2}+{\left(y-c_{new}\cdot y\right)}^{2}\leq r^{2}$


A pivot node is elected as the closest node to a center point. Replacement rule of the pivot node is not strictly defined and it could be determined according to sink group parameters e.g., region size or moving speed. We defined the rule in this paper as the time until a pivot belongs to the current group region for easy understanding and clear presentation. When the pivot calculates a new group region and the pivot is out of the region, it also delivers a *new_election* message when the new region is announced. Then, pivot $P_{2}$ is selected and announces the new region message with the new pivot information.

### 3.3. Guard Region

**Figure 5:**
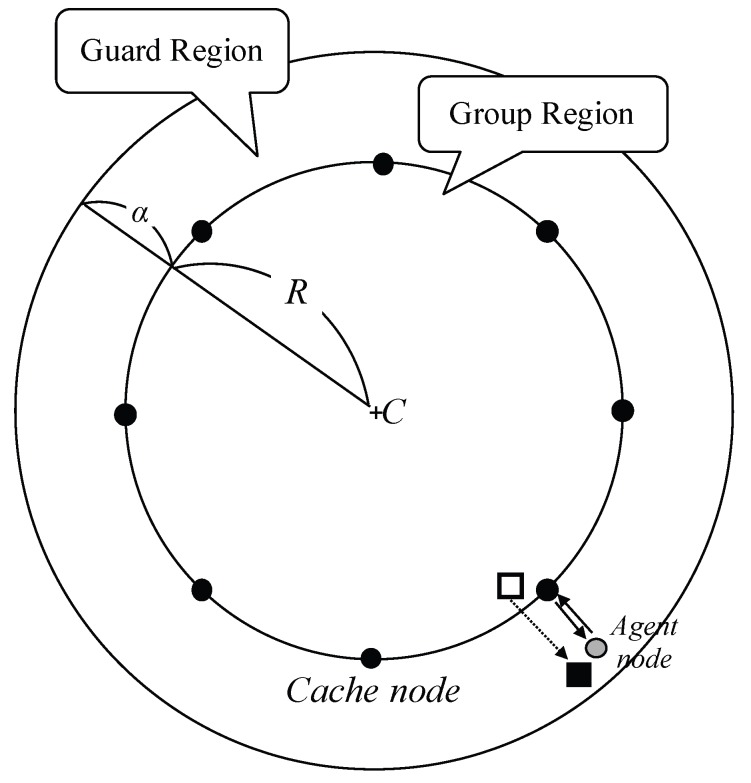
An example of a guard region and cache nodes.

Since we update region information by deviated member sinks, it may cause frequent updates rather than periodic ones in the case of fast varying sink group. In order to prevent the frequent updates, we introduce a supplement functionality, called the guard region. The guard region is defined as a circle also covering group region with radius R+α. If a member sink deviates out of the group region but exists in the guard region, the member sinks do not report to a pivot. Instead of that, cache nodes surrounding the group region support local movement of the sinks. Cache nodes are selected on eight sides as shown in Figure 5.

When a new region is updated, a previous pivot node includes positions of cache nodes. Note that the cache nodes are not explicitly designated. A member sink on the guard region selects an agent node and then it registers the member sinks to the closest cache position. The closest sensor node to the position keeps the information of the sink as a cache node and relays all packets in the group region via the agent. The cache node maintains its role until it receives a release message from the sink (when the sink re-enters into the region or re-registers to another close cache position) or new region information is updated.

### 3.4. Combining Deviated Location Information

**Figure 6:**
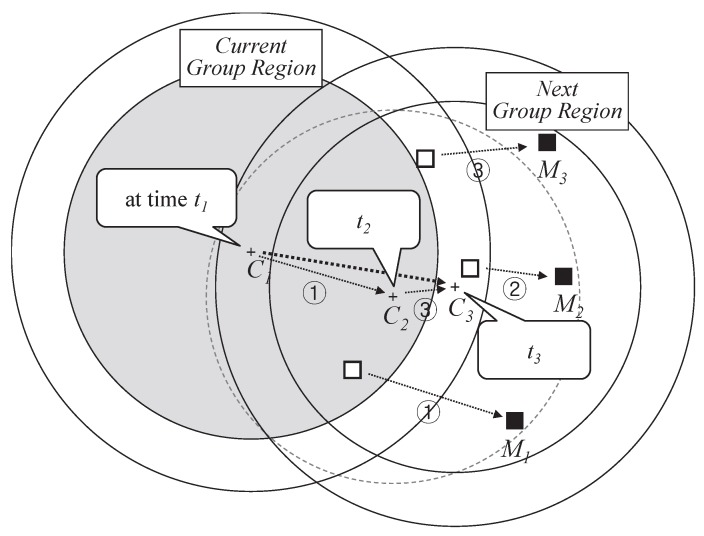
Combining locations of deviated sinks for interval time between data dissemination.

A *pivot* calculates a center point by combining information of member sinks out of the region until the next data packet reaches the *pivot*. As shown in Figure 6, there are three movements of member sinks. First, $M_{1}$ gets out of the region and the center point moves from $C_{1}$ to $C_{2}$ as much distance as the sink deviates. Next, $M_{2}$ moves at the time $t_{2}$. At this moment, $M_{2}$ knows that the location of the group is the location of ‘current group region’ so that $M_{2}$ recognizes being out of the group region. Then, $M_{2}$ reports its own location to the pivot. However, the pivot ignores the movement report of $M_{2}$ because it perceives that $M_{2}$ is not outside of the new region, the circle presented with gray dotted line. After that, $M_{3}$ deviates, and then the center point also moves to $C_{3}$. In this way, the new circle could hold all member sinks. In addition, moving direction of the group is adjusted from fragmentary information. If some sinks temporarily have a different direction from that of majority so that the region is shifted towards the direction, many deviation reports from the majority might occur and region is quickly adjusted. Even if all members have different directions from that of DGR, it does not violate mobility support since RSMGP still covers all member sinks as mentioned above in Figure 4.

## 4. Performance Evaluation

We compare the performance of our RSMGP with that of GMR [16], which is a traditional multicast scheme in WSNs and RingRouting [9], which is an efficient mobility support scheme for individual sinks. M-Geocasting [5] is compared as a primitive geocasting scheme to support mobile sink groups. In addition, we select ERMG [12] as the most recent study dealing with region discovery. We implemented three protocols in Network Simulator Qualnet Network Simulator (4.0, Scalable Network Technologies, Culver City, CA, USA) [17]. Sensor nodes follow the specification of MICA2 [18] and their transmission range is about 40 m. Simplified IEEE 802.15.4 was used as the PHY/MAC layer protocol. The size of the sensor network is set to 1500 m × 1500 m where 2500 nodes are randomly distributed. Initially, for simulation, the number of member sinks in a sink group is 10; the number of source node selected randomly is 20. Average energy consumption is defined as average consumption of twenty times of transmission. Transmitting and receiving power consumption rates of the sensors are 3.12 μJ and 2.34 μJ, respectively. The group moved following the *random waypoint* mobility model [19]. Default speed of the sink group is set to 3m/s and default radius of the group region is set to 100 m.

**Figure 7:**
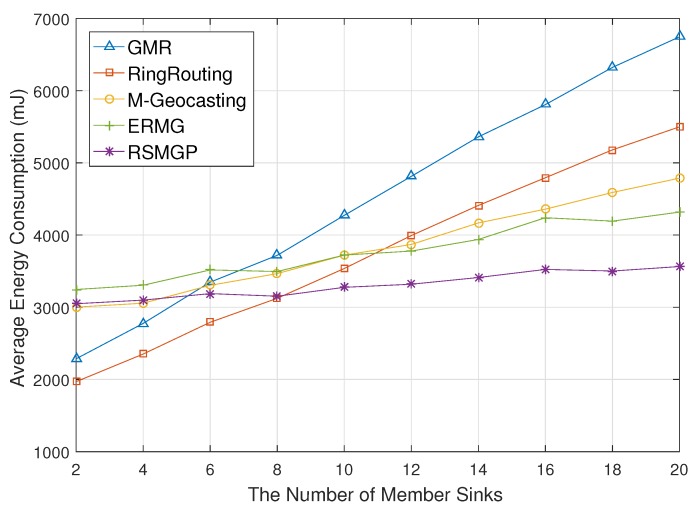
Energy consumption impacted by the number of member sinks.

Figure 7 shows average energy consumption for the number of member sinks of a group. If the number of member sinks increases, GMR consumes much energy in proportion to the number of sinks because they request all member sinks to report their location to the source. In RingRouting, although the ring structure rendezvous area effectively provides destination locations, the energy consumption increases due to individual location update and data dissemination. M-Geocasting consumes less energy for group location update than GMR since all member sinks do not need to update location to a source in M-Geocasting. ERMG also shows a similar tendency with M-Geocasting but with less energy consumption. The reason is that region discovery is conducted in the large expected area. The energy consumption of RSMGP does not significantly increase since our scheme calculates a group region using only a few member sinks and does not update the location.

**Figure 8:**
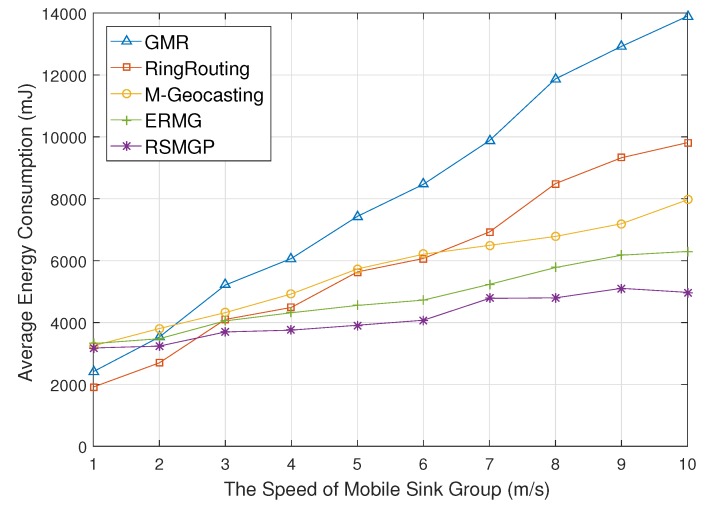
Energy consumption impacted by moving speed of a sink group.

Figure 8 presents average energy consumption for the speed of mobile sink group. If the speed increases, the energy consumption of both GMR and RingRouting increases due to frequent location updates to a source according to increasing movement speed. M-Geocasting consumes less energy than both schemes at high speeds because all member sinks do not need location updates to a source in M-Geocasting. However, as the speed increases, region discovery frequency by flooding becomes high. ERMG blocks frequent querying to some degree by cell unit communication. RSMGP efficiently supports the group mobility because it reduces calculation frequency and control messages.

**Figure 9:**
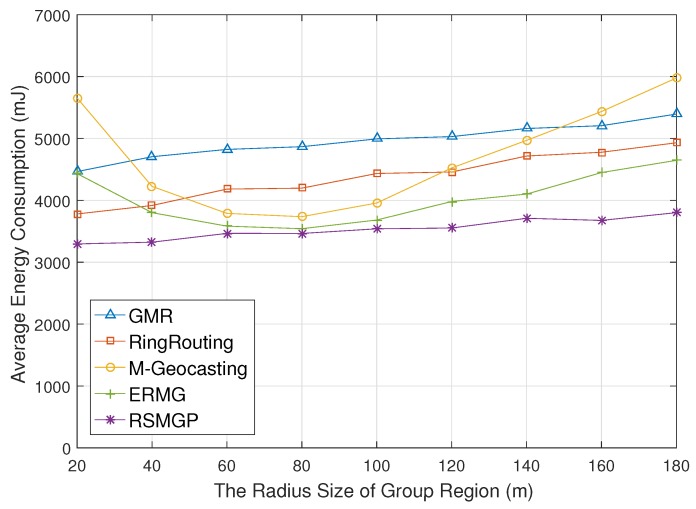
Energy consumption impacted by the radius of group area.

Figure 9 shows energy consumption for the radius of group area. In M-Geocasting and ERMG, the longer radius makes the mobile sink group stay in the calculated group region for a long time, but makes sensor nodes consume more energy for flooding. At first, therefore, energy consumption decreases until the radius is 100 m since recalculation rate for region decreases, but energy consumption rather increases as it increases the radius greater than 100 m due to flooding cost increasing rapidly. RSMGP, however, is not influenced by the radius of group region because only some sinks deviating group region is the factor that constructs the calculated group area. Consequently, in RSMGP, the energy consumption is almost similar regardless of the radius of group area as well as lower than that of M-Geocasting.

**Figure 10:**
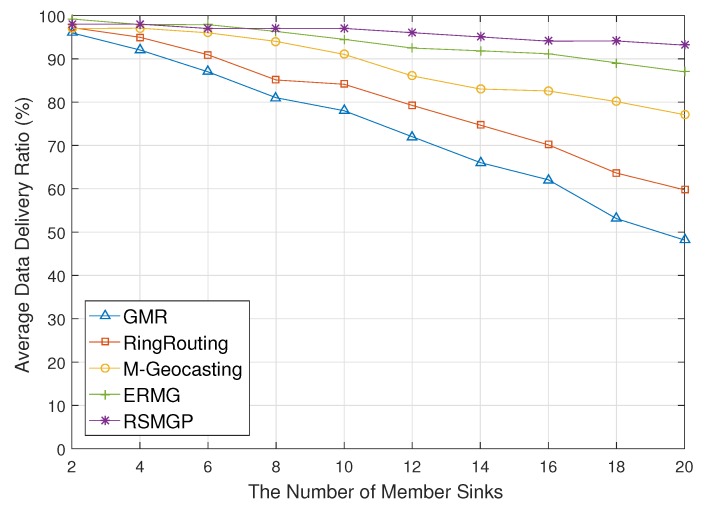
Data delivery ratio impacted by the number of member sinks.

Figure 10 illustrates data delivery ratio for the number of member sinks of a group. If the number of member sinks increases, average delivery ratio of GMR significantly decreases because concentration of packets around a source due to location updates of all member sinks causes congestion and collision. RingRouting also shows drop of the ratio due to the congestion of the rendezvous area. In M-Geocasting, if the number of member sinks grows, it increases loss of location report due to congestion and collision possibility around LS when LS obtains location information of member sinks. Therefore, region miscalculation may occur and drops data delivery ratio. ERMG shows a relatively stable ratio due to the fact that hierarchical communication reduces collision probability, but it still has a hot spot around cell heads. On the other hand, in RSMGP, it rarely takes effect on congestion and collision because only a few sinks deviate group region report location information to the pivot node.

**Figure 11:**
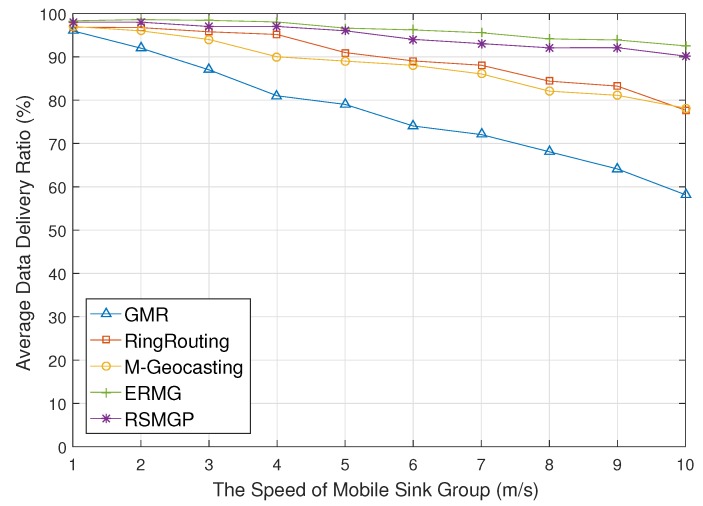
Data delivery ratio impacted by moving speed of a sink group.

Figure 11 depicts data delivery ratio for the speed of mobile sink group. If the speed increases, the data delivery ratio of GMR rapidly decreases. Every mobile sink in the group updates its own location to the source. Since all update messages are forwarded on a similar path, a number of message losses occur by collision and congestion. RingRouting prevents long-distance communication of the location update, but the congestion around rendezvous area drops the delivery ratio. In M-Geocasting, while it recalculates a group region and updates to a source, some sinks could not receive since group location information that sinks know may not exactly correspond with new information. Accordingly, the data delivery ratio of M-Geocasting decreases slightly as the speed of the group increases. However, in RSMGP, data are delivered with high delivery ratio since new location information of sink group is reflected directly by overhearing. In addition, ERMG is almost not influenced by the moving speed. The reason is that movement in a cell is affected only by its cell, and it does not cause long-distance communication and means low congestion.

**Figure 12:**
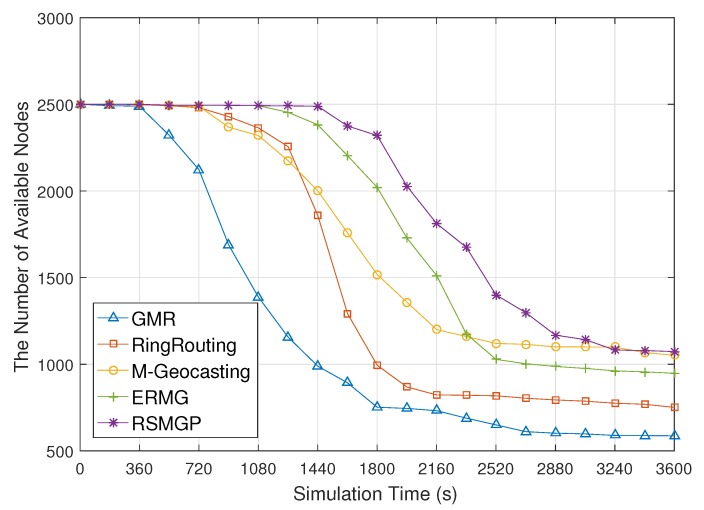
The number of available nodes according to elapsed simulation time.

Figure 12 shows the number of available nodes according to elapsed simulation time. In GMR, the nodes are depleted earlier than other schemes. This is because each of the member sinks updates its own location globally to a source and the traffic is concentrated around the source. In addition, as discussed above, the traffic concentration causes collision and congestion. Because of this, retransmission is frequently required and thus increases energy depletion more. RingRouting shows stable performance in the early stage, but the number of available nodes rapidly decreases due to detour paths after nodes around the rendezvous structure being depleted. On the other hand, M-Geocasting has one representative sink per group. Thus, the energy consumption of location update is trivial. However, region calculation based on flooding quickens the energy depletion of the nodes on the trajectory of a mobile sink group. ERMG reduces entire communication overhead, but it accelerates node depletion around cell heads. In RSMGP, only a few nodes participate in the region calculation and the intermediate nodes do not waste power on location updating. Thus, the scheme has an advantage over the network life time.

**Figure 13:**
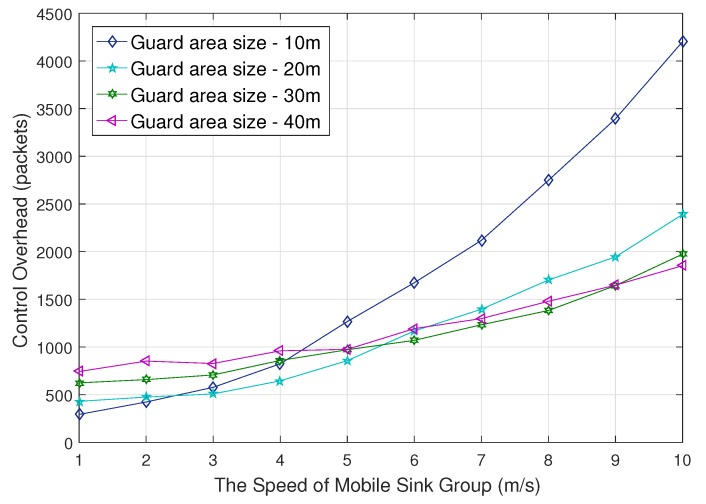
Control overhead impacted by guard area size.

Figure 13 compares the control overhead between various guard area size. We observe that small radius of the guard area is advantageous for caching, but is disadvantageous for region update. If the radius is too small, region departure reporting occurs too frequently in the case that a group moves fast. On the contrary, if the radius is too large, caching overhead increases in order to deliver data to member sinks in the guard area. Empirically, the range from 20 m to 30 m is considered to be appropriate according to this comparison, allowing practical movement speed ‘2 m/s–5 m/s’ for the applications discussed above.

## 5. Conclusions

In this paper, we propose a novel scheme for supporting mobile sink groups in wireless sensor networks. Our scheme avoids location updates of individual member sinks to a source in multicasting scheme, GMR. Different from M-Geocasting, since our scheme takes into account the inherent property of mobile sink group in which the movement of only sinks that deviate from the current sink group region reflects the movement of the mobile sink group, it calculates efficiently a group region with the location information of only the deviated member sinks instead of the location information of all member sinks. Thus, our scheme saves much energy through both low signaling overheads for calculating region of mobile sinks group. Simulation results show that our scheme consumes less energy than GMR and M-Geocasting.
